# Potential functions of embryonic cardiac macrophages in angiogenesis, lymphangiogenesis and extracellular matrix remodeling

**DOI:** 10.1007/s00418-020-01934-1

**Published:** 2020-11-01

**Authors:** Grzegorz Gula, Sławomir Rumiński, Justyna Niderla-Bielińska, Agnieszka Jasińska, Ewelina Kiernozek, Ewa Jankowska-Steifer, Aleksandra Flaht-Zabost, Anna Ratajska

**Affiliations:** 1 Postgraduate School of Molecular Medicine, Medical University of Warsaw, Warsaw, Poland; 2 Department of Ophthalmology, Central Clinical Hospital of the MSWiA, Warsaw, Poland; 3 Department of Histology and Embryology, Medical University of Warsaw, Warsaw, Poland; 4 Centre for Preclinical Research and Technology, Warsaw, Poland; 5 Department of Otorhinolaryngology, Head and Neck Surgery, Medical University of Warsaw, Student’s Scientific Circle at Department of Pathology MUW, Warsaw, Poland; 6 Department of Immunology, University of Warsaw, Warsaw, Poland; 7 Department of Pathology, Medical University of Warsaw, Chałubińskiego 5, 02-004 Warsaw, Poland

**Keywords:** Embryonic cardiac macrophages, Angiogenesis, Lymphangiogenesis, Extracellular matrix remodeling

## Abstract

The role of cardiac tissue macrophages (cTMs) during pre- and postnatal developmental stages remains in many aspects unknown. We aimed to characterize cTM populations and their potential functions based on surface markers. Our in situ studies of immunostained cardiac tissue specimens of murine fetuses (from E11to E17) revealed that a significant number of embryonic cTMs (phenotyped by CD45, CD68, CD64, F4/80, CD11b, CD206, Lyve-1) resided mostly in the subepicardial space, not in the entire myocardial wall, as observed in adult individuals. cTMs accompanied newly developed blood and lymphatic vessels adhering to vessel walls by cellular processes. A subpopulation of CD68-positive cells was found to form accumulations in areas of massive apoptosis during the outflow tract remodeling and shortening. Flow cytometry analysis at E14 and E17 stages revealed newly defined three subpopulations:CD64^low^, CD64^high^CD206-and CD64^high^CD206+. The levels of mRNA expression for genes related to regulation of angiogenesis (*VEGFa*, *VEGFb*, *VEGFc*, *bFGF*), lymphangiogenesis (*VEGFc*) and extracellular matrix (ECM) remodeling (*MMP13*, *Arg1*, *Ym1/Chil3*, *Retlna/FIZZ1*) differed among the selected populations and/or embryonic stages. Our results demonstrate a diversity of embryonic cTMs and their tissue-specific locations, suggesting their various potential roles in regulating angiogenesis, lymphangiogenesis and ECM remodeling.

## Introduction

Macrophages have been regarded as fundamental immune cells with phagocytic activity. Currently, however, macrophages started to be recognized by researchers as tissue-specific cells with novel and unexpected properties capable of performing various functions in physiological and pathological tissue processes (Gordon et al. 2014; Lavin et al. 2014; Leuschner and Nahrendorf 2020; Mass et al. 2016; Wynn et al. 2013). Also macrophage origins have been updated by discovering other sources of tissue macrophage besides from blood monocytes. Recent studies indicate that various populations of tissue resident macrophages are established during prenatal development originating from the yolk sac (from E7.0 to E11.5) and subsequently from hematopoietic stem cells (HSCs) in fetal liver (E11.5–E16.5) (McGrath et al. 2015; Schulz et al. 2012; Yona et al. 2013). Thus, macrophages start to colonize organs during early prenatal development (being first apparent within the heart about E9.5) and are gradually replaced by monocyte-derived macrophages and/or by local tissue proliferation in the later stages of development (from E17.5 in heart) and postnatally both in steady state and in disease (Davies et al. 2013; Epelman et al. 2014; Molawi et al. 2014). The rate and the time course of macrophage replacement are tissue-specific (Ginhoux and Guilliams 2016). Some exceptional populations of tissue macrophages such as liver Kupffer cells and brain microglia are of yolk sac origin persist in tissues into adulthood, whereas intestinal macrophages are replaced continuously during life. Discovering various sources of tissue macrophages began a new area of research, which is now focused more on seeking the origin–function relationship (Bajpai et al. 2018; Wang et al. 2020). Recently, the fetal endocardium lining endocardial cushions has been shown as another alternative independent source of embryonic cardiac macrophages. This subpopulation, characterized by high phagocytic activity, is necessary for normal valve development and contributes to valve pathologic remodeling (Shigeta et al. 2019). Moreover, some studies suggest that fetal cardiac macrophages represent a different phenotypic population compared with macrophage populations in other embryonic organs such as the lung, brain, liver, intestine, but still little is known about their possible functions. Embryonic cardiac tissue macrophages are identified as bearing F4/80^low^, CD11b^hi^, CX3CR1^high^ and CX3CR1^low^ markers. According to Epelman et al., the CX3CR1^high^ population is of yolk sac origin, whereas the CX3CR1^low^ is of fetal liver origin (Epelman et al. 2014).

There are a few theories on the possible signal pathways that regulate an invasion of the heart tissue by the yolk-sac-derived macrophages. One of these theories states that guiding macrophages into a specific niche in the subepicardial area is performed by the epicardium and its transcription factor Wilms tumor gene 1 (WT1) (Balmer et al. 2014; Stevens et al. 2016). Alternatively, another trigger for macrophage recruitment to the heart may come from the endothelium of newly developed coronary vessels via Notch signaling (Leid et al. 2016).

Some fetal cardiac macrophages were believed to be involved in coronary vessel development. Accordingly, Leid et al. proved that CCR2 macrophages, originating from the yolk sac, affect the remodeling but not the branching of the primitive subepicardial coronary vessel plexus. These macrophages could regulate coronary vessel expansion by a selective interaction with vessels at the onset of their perfusion with blood. This function is independent of the signals coming from the epicardium, pericytes and/or smooth muscle cells but is regulated by *IGF* signaling. In contrast, CCR2+ macrophages, derived from monocytes and recombination-activating gene 1 (Rag1) lymphomyeloid progenitors, were located close to the endocardium and their function during development still remains unclear (Leid et al. 2016; Wang et al. 2020).

The yolk-sac-derived embryonic macrophages revealed high proangiogenic and low proinflammatory potential in comparison with bone marrow-derived macrophages (Yosef et al. 2018). As both these subpopulations are present in the adult heart tissue, their roles and interplay could be crucial for heart function in physiologic and pathologic stages. In experimental studies, the self-renewable embryo-derived macrophages are essential for tissue repair and function restoration of the injured neonatal heart (Aurora et al. 2014). During the acute phase of myocardial infarction (MI) in the adult heart, resident macrophages recruit leucocytes (by secreting CXCL2 and CXCL5) and monocytes (by secreting CCL2) to the ischemic area. The pleiotropic roles of macrophages during the post-MI period include primarily phagocytosis as well as stimulation of other cells but it seems that many aspects remain unclear (Dick et al. 2019; Frantz and Nahrendorf 2014; Heidt et al. 2014; Honold and Nahrendorf 2018; Weinberger and Schulz 2015). Therefore, to better understand the role of cardiac tissue macrophages in different stages of heart disease, researchers often turn to prenatal processes, since the regulatory mechanisms of these processes are often very similar (Nucera et al. 2011).

In contrast to embryonic heart macrophages, adult heart macrophages have been studied more thoroughly. They constitute a multiphenotypic, phagocytic active population, whose gene expression profile resembles that of M2-type macrophages (Pinto et al. 2012; Van der Borght and Lambrecht 2018; Wang et al. 2020). Animal model findings are also reflected in human heart (Bajpai et al. 2018).

Epelman et al. proposed to divide the resident adult cardiac macrophages (F4/80+ CD11b+) into four subpopulations based on their origin and phenotypes. The most abundant population is of yolk sac origin and is capable of self-renewal. These cells are identified as CCR2^−^Ly6C^−^ with a marked differentiation on MHCII^high^ and MHCII^low^ subpopulations. The two other subpopulations (CCR2^−^Ly6C^+^ and CCR2^+^Ly6C^+^) come from the circulating monocytes and thus have HSC-derived precursors. Interestingly, during stress and pathological stages, circulating Ly6C^high^ monocytes could differentiate into all of these four subpopulations, which also gain the ability to self-renew (Epelman et al. 2014).

Currently, the classification of cardiac macrophages can be performed according to various criteria such as their origin, expression of specific combinations of markers, phenotypic and genetic profiles and functions. Macrophages are increasingly regarded as organ-specific cells and not only as a part of the immune system. Moreover, classification criteria might change with age (pre- and postnatally). Accordingly, we took aim to characterize cardiac tissue macrophages during heart development by specifying macrophage phenotypes and focusing on their putative function with the use of various methods. Three subpopulations of embryonic cardiac macrophages exhibited distinct phenotypes based on their possible proangiogenic, prolymphangiogenic and ECM remodeling functions as demonstrated by different gene expression profiles (different levels of selected mRNA molecules).

## Materials and methods

### Mice

This study was performed on an F1 cross of inbred C57BL/6 and CBA mouse strains. For immunofluorescence (IF) staining, murine hearts were taken from fetuses killed prenatally at different stages between E9.5 and E18.0. Hearts from E14 and E17 were obtained for flow cytometry and RT-PCR studies. Hearts from different litters (3–4 mice) were pooled to obtain a sufficient number of cells. For some control immunostainings, two postnatal and one adult (of 4-day-old and 10-week-old) mouse hearts were frozen and cryosectioned for assessment of the expression of certain immunomarkers (e.g., CD163-positive macrophage detection, myocardial location of CD45+, Lyve-1+, CD11b+, F4/80+, CD206+ cells).

### Immunofluorescence staining and confocal microscopic analysis

Multiple-color immunohistochemical staining was performed both on embryonic heart tissue cryosections and on whole-mount-stained hearts to analyze these specimens Leica inverted microscope [DMI6000-CS, Leica Microsystems, Germany, model—Leica TCS SP5, equipped with LAS AF software; objectives used: HCX PL APO CS 10.0 × 0.40 DRY UV (11506285) HCX PL APO lambda blue 20.0 × 0.70 IMM UV (11506191)].

Hearts were excised from fetuses between E9.5 and E18 and harvested in a sterile Tris-Tyrode solution. For cryosections, hearts were immediately frozen in liquid nitrogen after mounting in Tissue Freezing Medium (Leica Microsystems). Sections (10 µm thick) were cut serially in a cryostat, fixed in fresh 4% paraformaldehyde (PFA)/PBS for 30 min, washed with PBS for 2 × 10 min and incubated with 1% BSA/0.1% TritonX-100/0.1 M glycine/PBS for 30 min and then washed with PBS 2 × 10 min. The blocking step with specific serum was omitted (Buchwalow et al. 2011). One-hour incubation with various combinations of the primary antibodies, listed in Table 1, was performed. After washing with PBS (2 × 10 min), secondary antibodies, also listed in Table 1, were added for 1 h. At the end, the sections were washed with PBS (2 × 10 min) and counterstained with DAPI (Thermo Fisher Scientific, MA, USA) according to the manufacturer’s protocol and finally were mounted in fluorescence-mounting medium (Dako, Carpinteria, CA, USA).

**Table 1:** Primary and secondary antibodies used in immunofluorescent staining

Primary antibodies	Supplier	Host	Dilution	Catalog number
Caspase-3	Cell Signaling Technology, Danvers, MA, USA	Rabbit	1:50	9664
CD11b	BD Biosciences, San Jose, CA, USA	Rat	1:30	550282
CD163	Santa Cruz Biotech., Dallas, TX, USA	Rabbit	1:50	sc-33560
CD206	Invitrogen, Carlsbad, CA, USA	Rat	1:100	MA5-16871
CD31	BD Biosciences	Rat	1:100	550274
CD34	Biorbyt, Cambridge, UK	Rabbit	1:50	orb27549
CD45	BD Biosciences	Rat	1:50	550539
CD64	Bio-Rad, Hercules, CA, USA	Rat	1:800	MCA5997
CD64	Santa Cruz Biotech	Goat	1:50	sc-7642
CD68	Abcam, Cambridge, UK	Rat	1:400	ab53444
CD68	Abcam	Rabbit	1:25	ab125212
F4/80	Invitrogen	Rat	1:400	14-4801
Flk1	R&D Systems, Minneapolis, MN, USA	Goat	1:45	AF644
Lyve-1	Angiobio Co., Del Mar., CA, USA	Rabbit	1:100	11-034
Lyve-1	R&D Systems	Rat	1:300	MAB2125
Prox-1	Angiobio	Rabbit	1:60	11-002
WT1	Dako	Mouse	1:30	M3561
Zeb2	Invitrogen	Rabbit	1:100	PA5-20980
Others				
GSI	Sigma-Aldrich, St. Louis, MO, USA	TRITC conjugated	1:100	L5264
WGA	Invitrogen	Alexa Fluor 488 conjugated	1:1800	W11261

Secondary antibodies (Jackson ImmunoResearch Lab., West Grove, PA, USA)	Host	Dilution	Catalog number
Donkey anti-rabbit Cy™3 IgG (H + L)	Rabbit	1:800	711-165-152
Donkey anti-rat AlexaFluor 647 IgG (H + L)	Rat	1:500	712-605-153
Donkey anti-goat FITC IgG (H + L)	Goat	1:200	705-095-147
Donkey anti-mouse FITC IgG (H + L)	Mouse	1:200	715-095-150

For whole-mount embryonic heart specimens (up to 200 µm thick) were treated according to the protocol by Mukouyama, with our own modifications (Mukouyama et al. 2012). Immediately after excision of embryonic hearts, they were divided using microscissors into two pieces in frontal plane, rinsed from blood cells and fixed in fresh 4% PFA/PBS overnight. Then, the specimens were washed with PBS (2 × 20 min), incubated with 1% BSA/0.1%TritonX-100/0.1 M glycine/PBS for 30 min and thoroughly rinsed with PBS as above. Then, the blocking of nonspecific background staining with 10% donkey serum (Jackson ImmunoResearch Laboratories, USA) was performed for 1 h. Subsequently, heart specimens were incubated overnight with a mixture of primary antibodies (Table 1). The following steps were exactly as with cryosections, except the washing steps in PBS were 1 h each and incubation with secondary antibodies lasted 5 h.

Whole-mount staining was performed also on paraformaldehyde‐fixed fetal hearts according with the protocol preciously described in our previous studies using anti-F4/80, rat and/or anti-CD68, rabbit antibodies (Table 1) (Flaht et al. 2012; Jankowska-Steifer et al. 2015).

### Isolation of single cells and flow cytometry analysis

Hearts dissected from E14- and E17-stage fetuses were flushed with DMEM containing 1% antibiotic/antimycotic solution and cut with microscissors under a stereomicroscope control into small pieces to efficiently rinse out the blood cells. These parts of hearts containing ventricles, atria with auricles and short fragment of the outflow tract were digested with Accutase (Sigma-Aldrich; St. Louis, MO, USA). At least 20–23 fetal hearts were pooled from 2–3 litters of the same age per one run of the experiment. After mixing gently on a magnetic stirrer in Accutase solution for 15 min, the cell suspension was pipetted up and down and then digestion in Accutase with stirring was repeated. The dissociated cells were passed through 40-μm mesh filter, pelleted by centrifugation, washed with PBS twice, counted and incubated in fresh FACS buffer (PBS/1% BSA) for 20 min. Afterward, primary antibodies were added to each sample for a 30-min incubation at room temperature in the dark. The panel of selected antibodies included CD11b-PE-Cy7, CD45-BV510, F4/80-BV421, CD64-AF647 (BD Biosciences, Pharmingen, San Diego, CA, USA); Lyve-1-PE, CD206-PerCP, CX3CR1-AF488 (R&D Systems, Minneapolis, MN, USA). The eBioscience™ Fixable Viability Dye eFluor™ 780 (Invitrogen, Carlsbad, CA, USA) was used as viability control. Subsequently, the cells were washed once with a FACS buffer and resuspended in a fresh buffer with 1% PFA. Samples were stored at 4 °C, and the analysis was performed within 72 h of staining using a BD FACSCanto™ II cytometer (BD Biosciences, Pharmingen, San Diego, CA, USA. Immediately before the analysis, FACS calibration beads were stained with the antibodies and were analyzed to compensate for spectral overlap between the various fluorochrome channels. Data analysis was performed on Diva Software and Flowing Software.

### Cells sorting by flow cytometry and PCR analysis

The process of obtaining single cell suspension and performing immunostaining for flow cytometry was the same as described above. Immediately after staining and rinsing in FACS buffer twice, the cells were analyzed and sorted by BD FACSAria™ III sorter (BD Biosciences, Pharmingen, San Diego, CA, USA. RNA from sorted subpopulations of macrophages was isolated using a PureLink™ RNA Micro Scale Kit (Invitrogen, Carlsbad, CA, USA) according to the producer’s protocol. Concentration and quality of RNA were assessed with a NanoDrop spectrophotometer. The obtained RNA was transcribed into cDNA according to the manufacturer’s protocol (High-Capacity cDNA Reverse Transcription Kit, Thermo Fisher Scientific). Relative quantification of gene expression was performed using Real-time PCR at Abi Prism 7500 (Thermo Fisher Scientific) in 96-well optical plates. Endogenous control (mouse GAPDH: Mm99999915_g1) was used during triplication of each sample. To detect mRNA levels of selected genes, the following TaqMan Gene Expression Assays were used: *MMP13*: Mm00439491_m1; *VEGFa*: Mm01281449_m1; *Fgf2*: Mm00433287_m1; *IL-10*: Mm00439614_m1; *Chill3*: Mm00657889_mH; *Arg1*: Mm00475988_m1; *Retnla*: Mm00445109_m1; *Igf1*: Mm00439560_m1; *VEGFc*: Mm00437310_m1; and *VEGFb*: Mm00442102_m1. The reactions were performed typically, in a 25 μl volume with TaqMan Universal PCR Master Mix (Thermo Fisher Scientific), primers set, a MGB probe and 5 ng of cDNA template (previously stored at − 20 °C). The thermal conditions were: 10 min at 95 °C and 40 cycles of 15 s at 95 °C and 1 min at 60 °C. Sequence detection software v1.2 (Thermo Fisher Scientific) was used for analysis.

For RT-PCR data, The Mann–Whitney U/Wilcoxon test was applied using Statistica software v12 (StatSoft, Tulsa, USA). Differences were considered statistically significant with *p* value < 0.05. The values displayed represent mean ± SEM, *n* = 5 per group.

## Results

### Location of embryonic cTMs

**Fig. 1:**
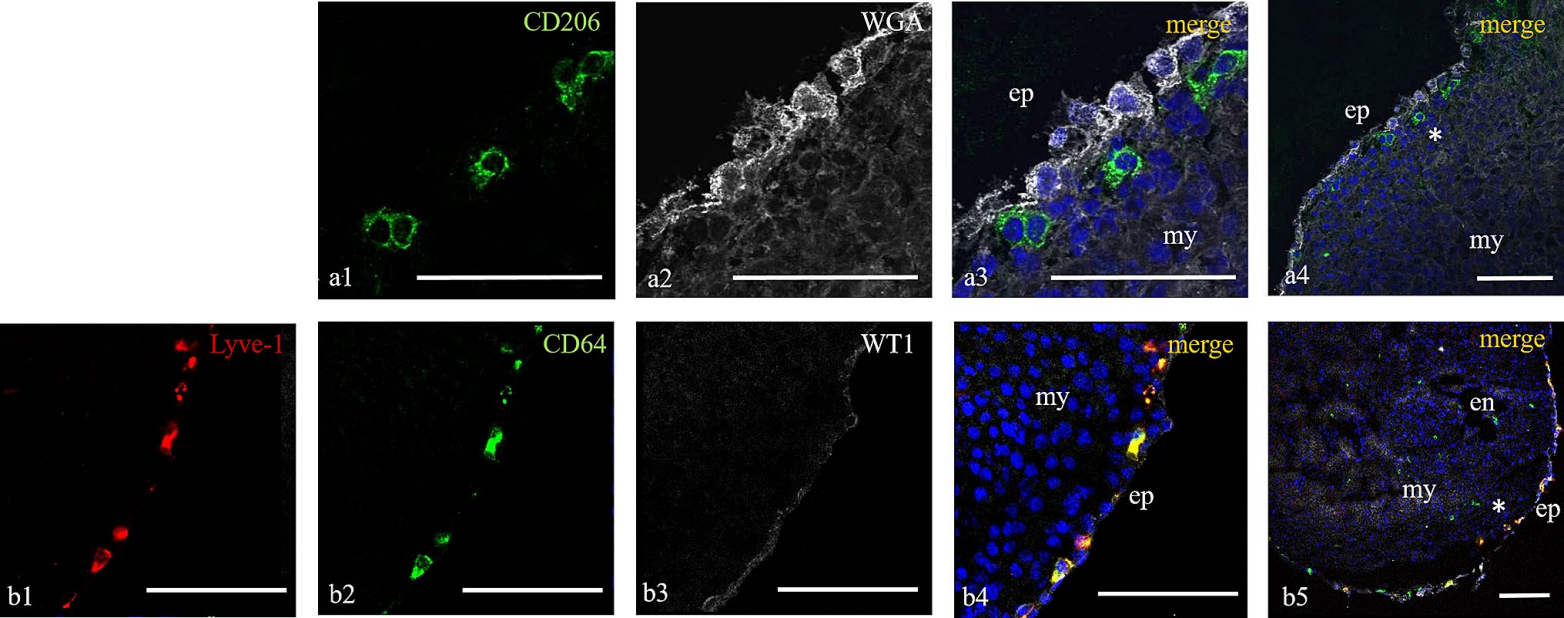
Subepicardial location of embryonic macrophages. Cross sections at the level of mid-ventricle of E16 (**a1**–**a4**) and E12 (**b1**–**b5**) hearts. The epicardium is labeled with WGA lectin (**a2**–**a4**) and with WT1 antigen (**b3**–**b5**), and less intense staining with WGA is visible around cardiac cells (**a2**–**a4**). The CD206-positive (**a1, a3,** **a4**) and the Lyve-1+/CD64+ macrophages are located under the epicardial cells (**b1**–**b5**); asterisks in panels a4 and b5 indicate the magnified area in **a1**–**a3** and **b1**–**b4**, respectively. *WGA* wheat germ agglutinin, *WT1* Wilms’ tumor protein, *ep* epicardium, *my* myocardium, *en*—endocardium; scale bars—100 µm

Confocal microscopic studies on cryosections revealed a large number of hematopoietic cells (HCs) bearing the CD45 antigen scattered predominantly in the subepicardial area. The epicardium was identified by WT1, WGA lectin (Wheat Germ Agglutinin) or Zeb2 markers for proper anatomical orientation of cardiac tissue (Fig. 1). In previous studies, we observed that the first WT1+ cells of the proepicardial organ started to cover the myocardial surface about E9.5–10.5 and gave rise to the epicardium (Niderla-Bielinska et al. 2015). Soon after that, the first CD45+WT1-Zeb2-HCs were detected subepicardially. Most of these cells bore also the following antigens: F4/80, CD68, CD64, CD206, Lyve-1 and CD11b (a myeloid cell marker) and therefore were identified as cardiac tissue macrophages (cTMs) (Fig. 1). Of note, none of these single scattered cells expressed either Flk-1, a hemangioblast and endothelial cell marker, or the CD163 antigen, which is a high-affinity scavenger receptor, detected in neonatal and adult cardiac macrophages.

Originally present in the subepicardium, macrophages spread gradually throughout the heart tissue during development invading deeper layers of the myocardial wall by the postnatal age and in adults.

**Fig. 2:**
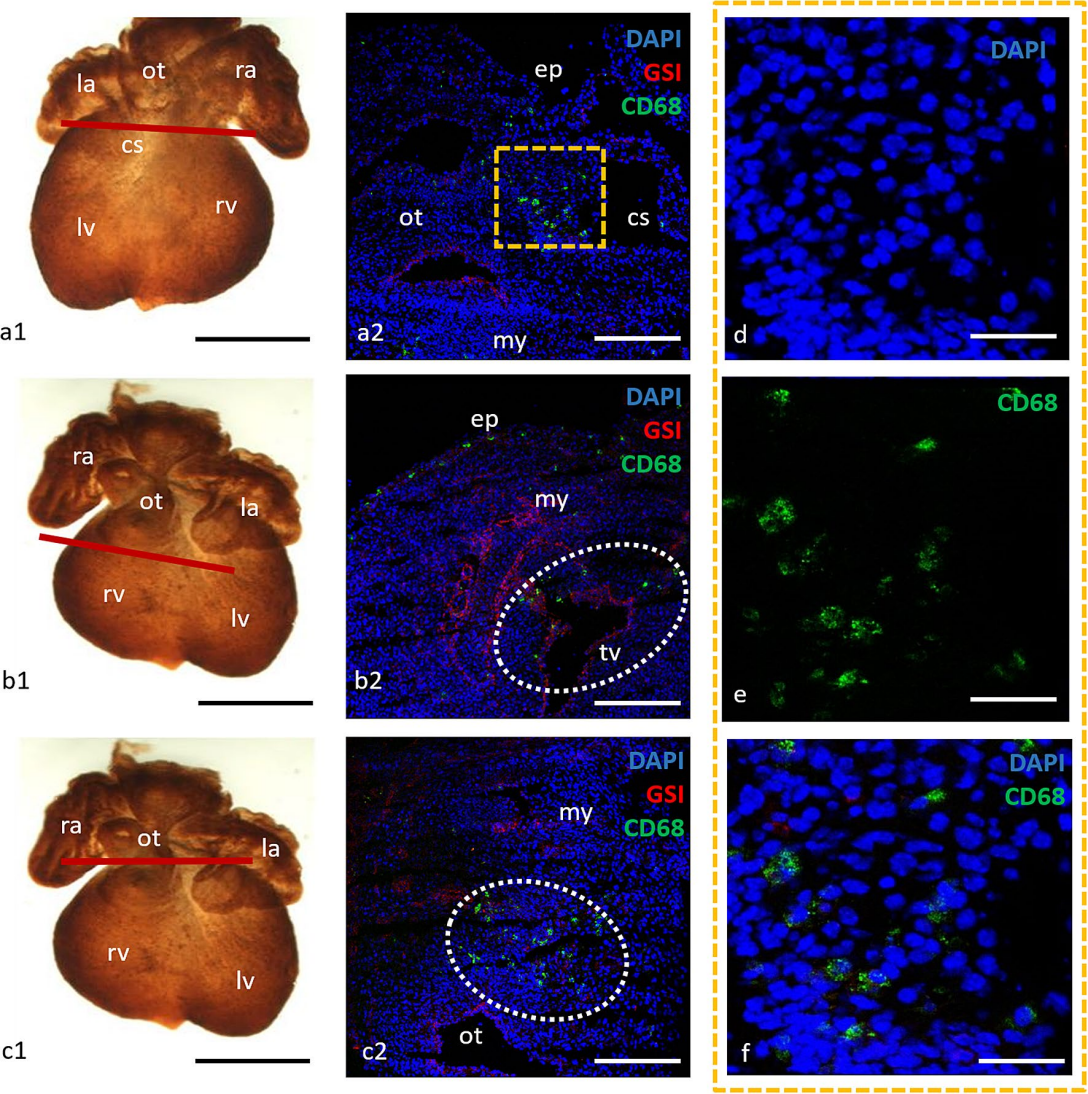
CD68 + macrophages are present in the apoptotic areas during heart development. Whole-mount immunostained specimens of E13.0 hearts (**a1**–**c1**). Red line indicates the levels of tissue sections. Immunostaining (**a2**–**c2**) of cryosections of E13 hearts analyzed in a confocal microscopy. The subpopulation of cTMs, marked CD68+, is specifically located in the apoptotic zones (dashed and dotted areas) during heart development, i.e., the area of coronary sinus (**a1**, **a2**); the area of developing tricuspid valve (**b1**, **b2**) and the ventricular outflow tract (**c1**, **c2**). Magnification of the dashed line marked area in a2 is shown in the marked right panel (**d**–**f**). *GSI* Griffonia Simplicifolia lectin I marks endothelial and endocardial cells, *la* left atrium, *lv* left ventricle, *ra* right atrium, *rv* right ventricle, *ot* outflow tract, *cs* coronary sinus, *ep* epicardium, *my* myocardium, *tv* tricuspid valve. Scale bars: **a1**–**c1**—400 µm; **a2**–**c2**—100 µm; **d**–**f**—50 µm

Interestingly, at stages E12–13, a high density of cTMs (bearing the CD45+CD68+Lyve-1−CD31−GSI− phenotype) was observed in apoptotic-body-rich areas, identified with DAPI and confirmed by anti-caspase-3 staining (Doonan and Cotter 2008). These areas correspond to remodeling/shortening of the outflow tract, remodeling of the interatrial septum and developing atrioventricular valves (Fig. 2).

### Link between embryonic cTMs and newly formed vessels.

**Fig. 3:**
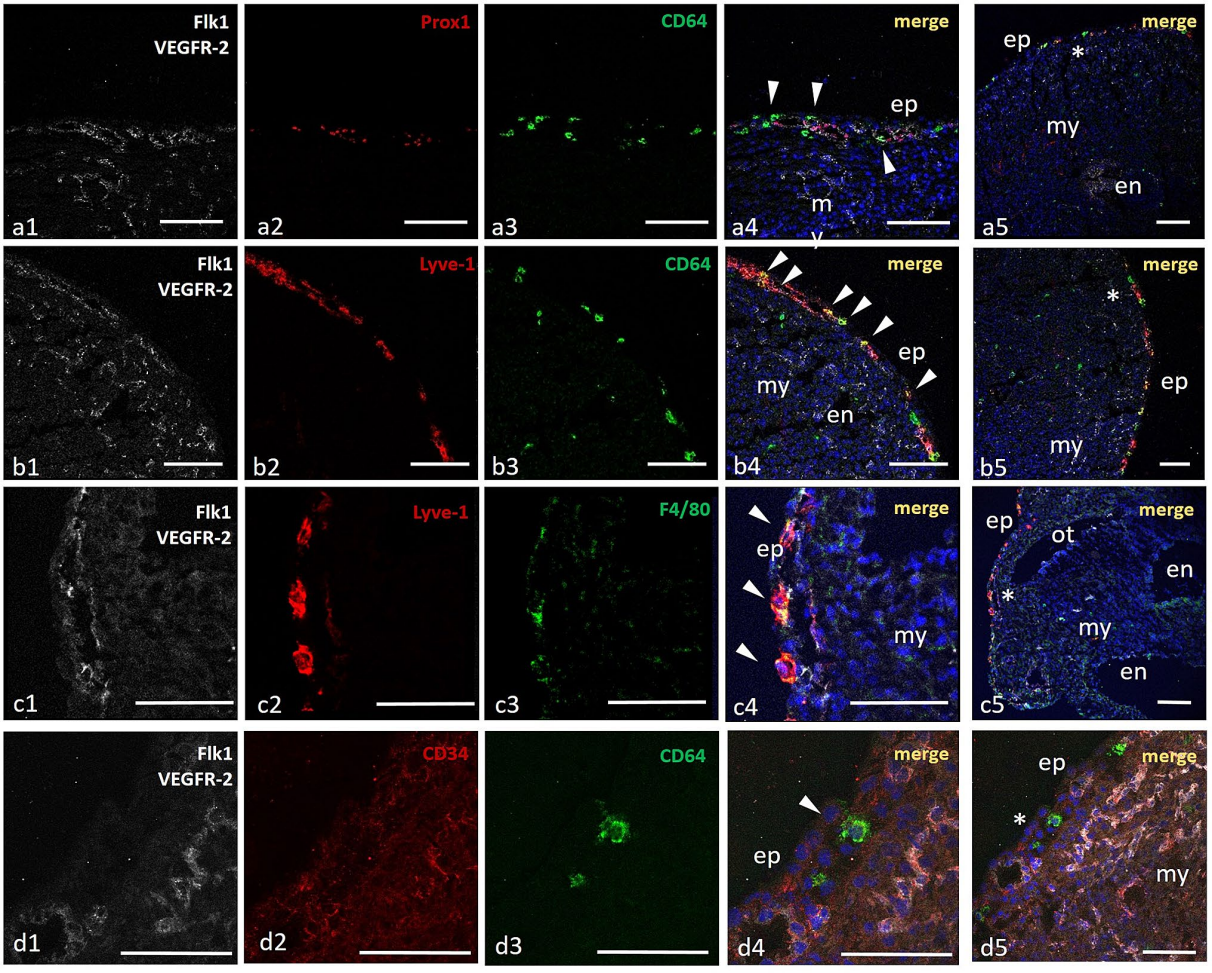
Embryonic cTMs adhere to newly developed lymphatics and veins. Cryosections of E13.0 murine hearts at the level of mid-ventricles immunostained for CD64 and F4/80 macrophage antigens. CD64+ and F4/80+ cells adhere to walls of newly developed lymphatics (Flk1+/Prox1+) — **a1**–**a4**, and initial veins (Flk1+, Lyve-1+) — **b1**–**b4** and **c1**–**c4**, are located in the vicinity of Flk1/CD34 double-positive endothelial cells of blood vessels — d1–d4. Single scattered cTMs are also positive for Lyve-1 (**b4**, **c4**), but remain Flk1−; CD34− and Prox1-negative. Single scattered CD64+ cells are detected also in myocardium (**b3**). *ep* epicardium, *my* myocardium, *en* endocardium, *ot* outflow tract; asterisks in **a5**–**d5** indicate the magnified area in **a1**–**a4**, **b1**–**b4**, **c1**–**c4** and **d1**–**d4**, respectively. Arrowheads indicate macrophages. Scale bars—100 µm

In situ studies demonstrated that all phenotypically defined subpopulations of cTMs were positioned in the vicinity of newly developed blood and lymphatic vessels (Fig. 3). Triple IF staining of serial sections for Flk1 (VEGFR2), Prox1 and Lyve-1 resulted in detection of newly formed coronary capillaries (Flk1+Lyve-1−Prox1− or Flk1+CD34+), veins (Flk1+Lyve-1+Prox1− or Flk1 + CD34 +) and lymphatic vessels (Flk1+Lyve1+Prox1+ or Flk1+ CD34−). At E11–12 stages cTMs were predominantly located in the vicinity of these parts of vessel walls that were adjacent to the epicardial surface (Fig. 3) Although cTMs were often coopted to vessel walls, they did not gain endothelial antigens such as Flk1, CD31 (PECAM-1), Prox-1, VEGFR3, CD34 or Tie2.

### Heterogeneity of cTMs during heart development

**Fig. 4:**
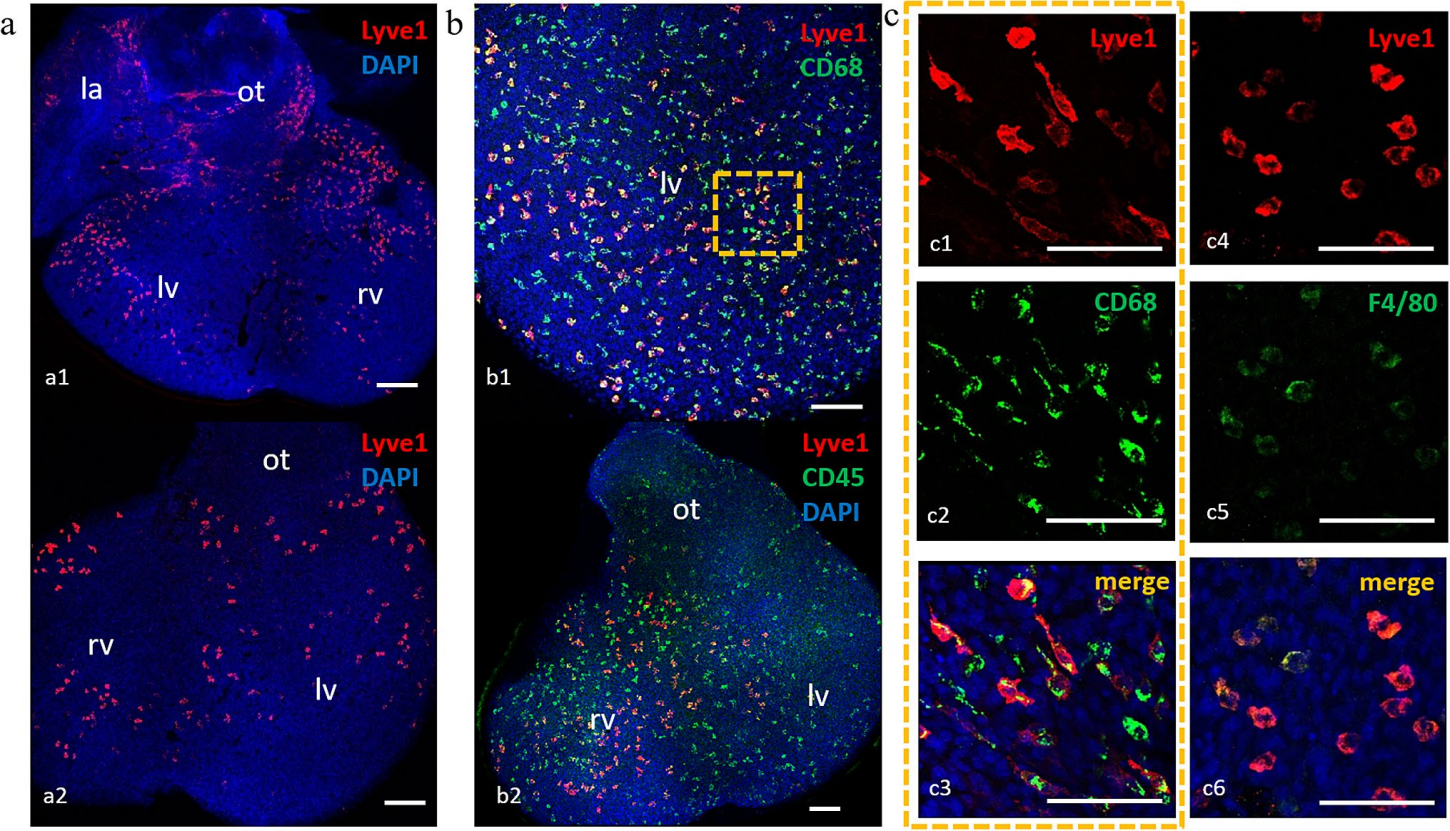
Heterogeneity of embryonic cTM phenotypes in a confocal microscope. Whole-mount immunostained murine heart specimens of E12.5–13.0. **a** Lyve-1+ single scattered cells are located on the myocardial surface on the both sides of heart: ventral (above) and dorsal (below). Lyve-1 cells are visible on the whole heart surface, with a decreasing density from the heart base (the outflow tract) to the heart apex. Lyve-1+ cells settle the subepicardial area from about E11.5, and their number increases with heart development, as shown in our previous study (Flaht et al, 2012). Lyve-1 cells could be easily detected on several z-stacks of the heart surface scans. **b** Merged images showing co-staining of Lyve-1 with CD68 and/or CD45 in a representative Z-scan taken at the level of heart surfaces. The following populations, covering the entire heart surface, are visible: CD68+/Lyve1+, CD68+/Lyve-1−, CD45+/Lyve-1+, and CD45+/Lyve1−. None of these cells exhibits CD45−/Lyve-1+ or CD68−/Lyve-1+ phenotype confirming that Lyve-1+ cells possess a hematopoietic origin, as CD45 + cells likely represent CD68-positive macrophages. **c** Magnification of the dashed line-marked area in **b1** is shown in the right panels (**c1**–**c3**). These panels show a Z-scan taken at the level of heart ventricle surfaces. The following subpopulations of macrophages are detectable: CD68+/Lyve1+; CD68+/Lyve-1− (**c1**–**c3**), and F4/80+/Lyve-1+ (**c4**–**c6**). *la* left atrium, *lv* left ventricle, *rv* right ventricle, *ot* outflow track; scale bars − 100 µm

Macrophage subpopulations differ with respect to the expression of various markers, as assessed in a confocal microscope of immunostained whole-mount specimens and cryosections. Whole-mount method immunostaining revealed subepicardial location of cTMs (Fig. 4a). A considerable number of single scattered subepicardial Lyve-1+ cells expressed CD45, an immune cell marker (Fig. 4b2). Lyve-1+ cells expressed also the CD68 and/or the F4/80 antigens and, therefore, could be classified as cTMs (Fig. 4b1, c). These Lyve-1+/CD68+ and Lyve-1+/F4/80+ cells did not gain endothelial markers (Flk-1, CD31) during heart development. This distinguished them from Lyve-1+ cells of lymphatic vessel endothelium and potential lymphangioblasts which possessed Flk-1 and CD31 antigens (which is consistent with the observation by Stevens et al. 2016). Among macrophages, the following subpopulations could be detected: CD68+Lyve-1+ and CD68+Lyve-1−; F4/80+Lyve-1+ and F4/80+Lyve-1−.

### Seeding of fetal heart with cTM

**Fig. 5:**
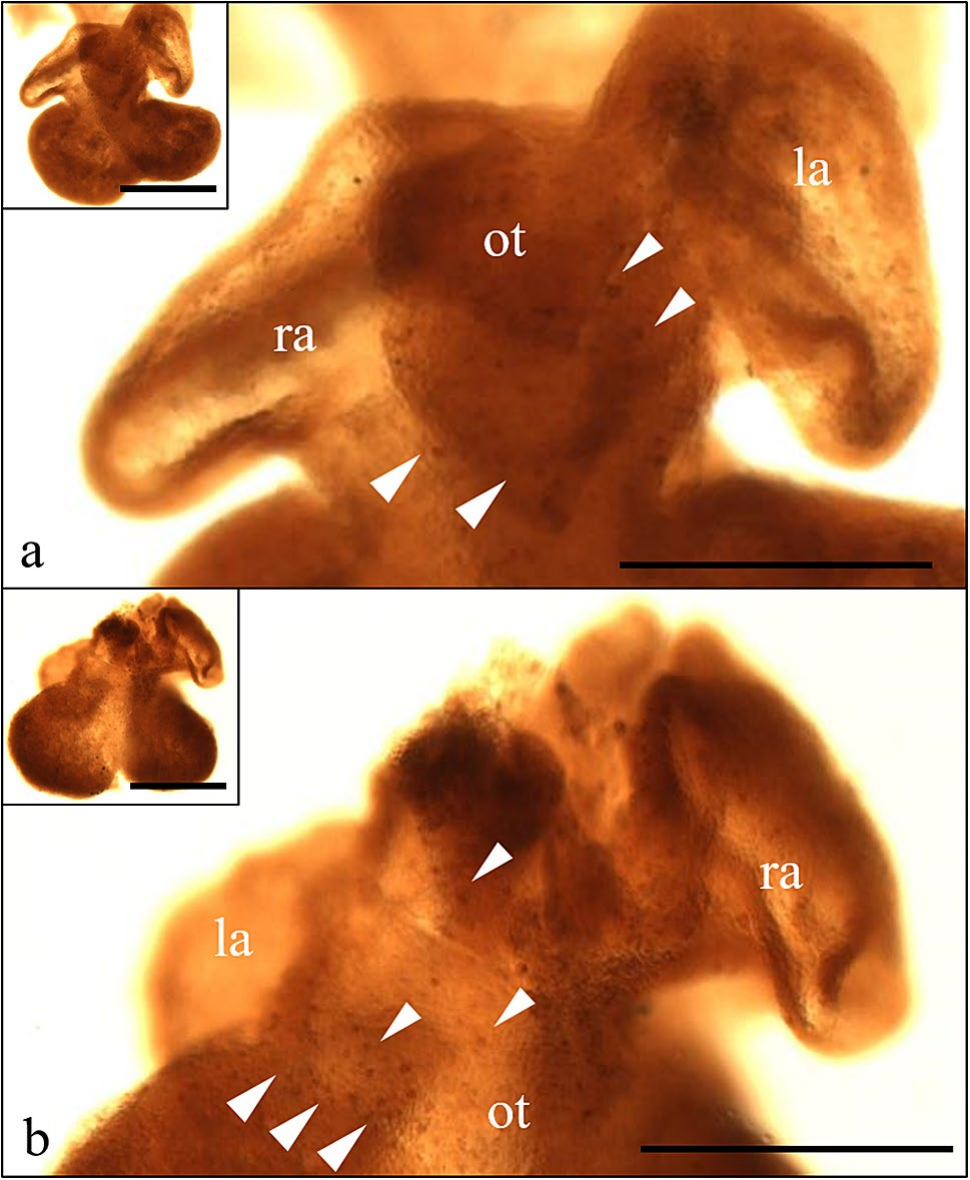
First F4/80+ cells detected within heart. Whole-mount immune-stained murine hearts (E10) with anti-F4/80 antibodies. The first single F4/80+ cells (marked with arrowheads) are detected on the outflow tract (**a**) and atrial (**b**) heart surfaces. *la* left atrium, *ra* right atrium; *ot* outflow tract. Scale bars—500 µm

The first CD68+ and F4/80+ cells were detected on the ventral and the dorsal surfaces of the outflow tract and in dorsal atrioventricular sulcus at E10 (Fig. 5). Then, CD68+F4/80+ cell expansion was found to be colocalized with the areas of coronary and lymphatic plexuses development, whose progression, originally subepicardial, was subsequently directed toward myocardium. At E12.5–E13, macrophages covered the whole atrial and ventricular surfaces (Fig. 4b). While development proceeds, the density of macrophages increases.

### Macrophage–endothelium interplay

**Fig. 6:**
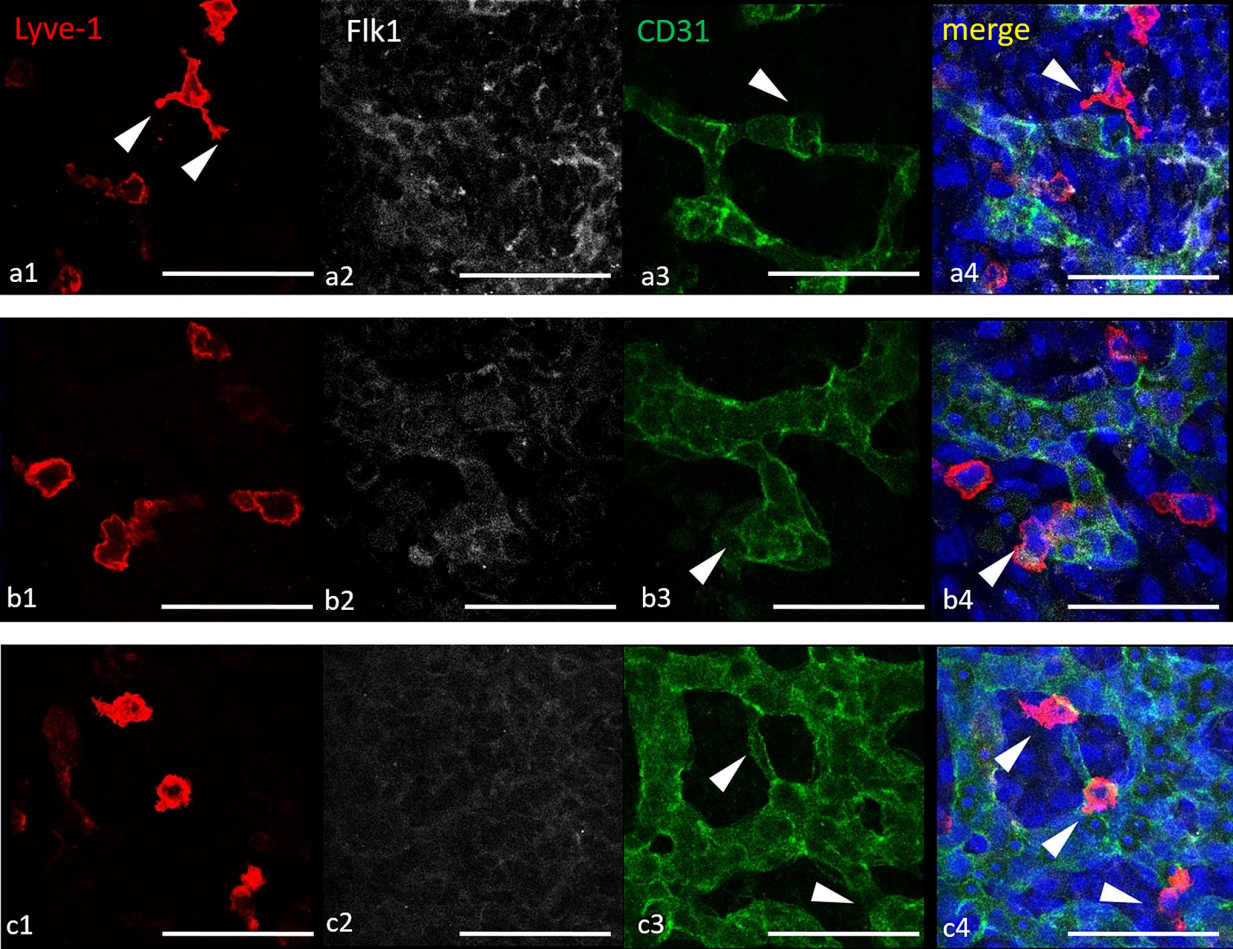
Morphology and colocalization of Lyve-1+ macrophages and endothelial cells. Murine hearts of E13 whole-mount immunostained to endothelial cell-specific and Lyve-1 antigens. Representative Z-scans taken at the level of heart surfaces from mid-ventricles. Colocalization of single Lyve-1+ cells with newly formed cardiac vessels identified by Flk1+ (VEGFR2+)/CD31+ endothelial cells. The amoeboid shape of Lyve-1+ macrophages with protrusions is marked with arrowheads (**a1, a4**). Lyve-1+ macrophages are localized adjacent to bridging tips of new vessels (**a3**, **a4**, arrowheads), sprouts (**b3**, **b4,** arrowheads) or branches (**c3**, **c4**, arrowheads). Scale bars—100 µm

The assessment of whole-mount immunostained specimens showed Lyve-1+ cells in the subepicardial area. Some of them took an amoebic shape with finger-like projections, as typical phagocytic macrophages (Fig. 6a). Moreover, most of Lyve-1+ cells colocalized with newly formed capillaries (of CD31+/Flk1+ phenotype) (Fig. 6a–c). We detected macrophage interaction with the tips of endothelial cells (Fig. 6a) as well as with the sprouts (Fig. 6b) and bridges/branches between neighboring newly formed vessels (Fig. 6c). These in situ findings seem to support our hypothesis about cTM role in vessel biology/angiogenesis/vessel wall patrolling (stabilization of a vessel wall), etc.

### Identity (classification) of novel embryonic cTM subpopulations.

FACS analysis helped identify both the set of markers expressed on different subpopulation of macrophages and the number of macrophages within selected cTM populations in E14 and E17 hearts. Since circulating blood cells were present in freshly harvested heart specimens (within the chambers and coronary vessel lumina) and therefore could be a potential source of macrophages or monocytes, we also performed control FACS analyses (in triplicate) of blood cells obtained from fetuses. This study revealed that the number of immune cells (CD45+CD11b+) detected in blood samples was negligible compared with the number of the same population obtained from single cell suspension of fetal hearts (data not shown).

**Fig. 7:**
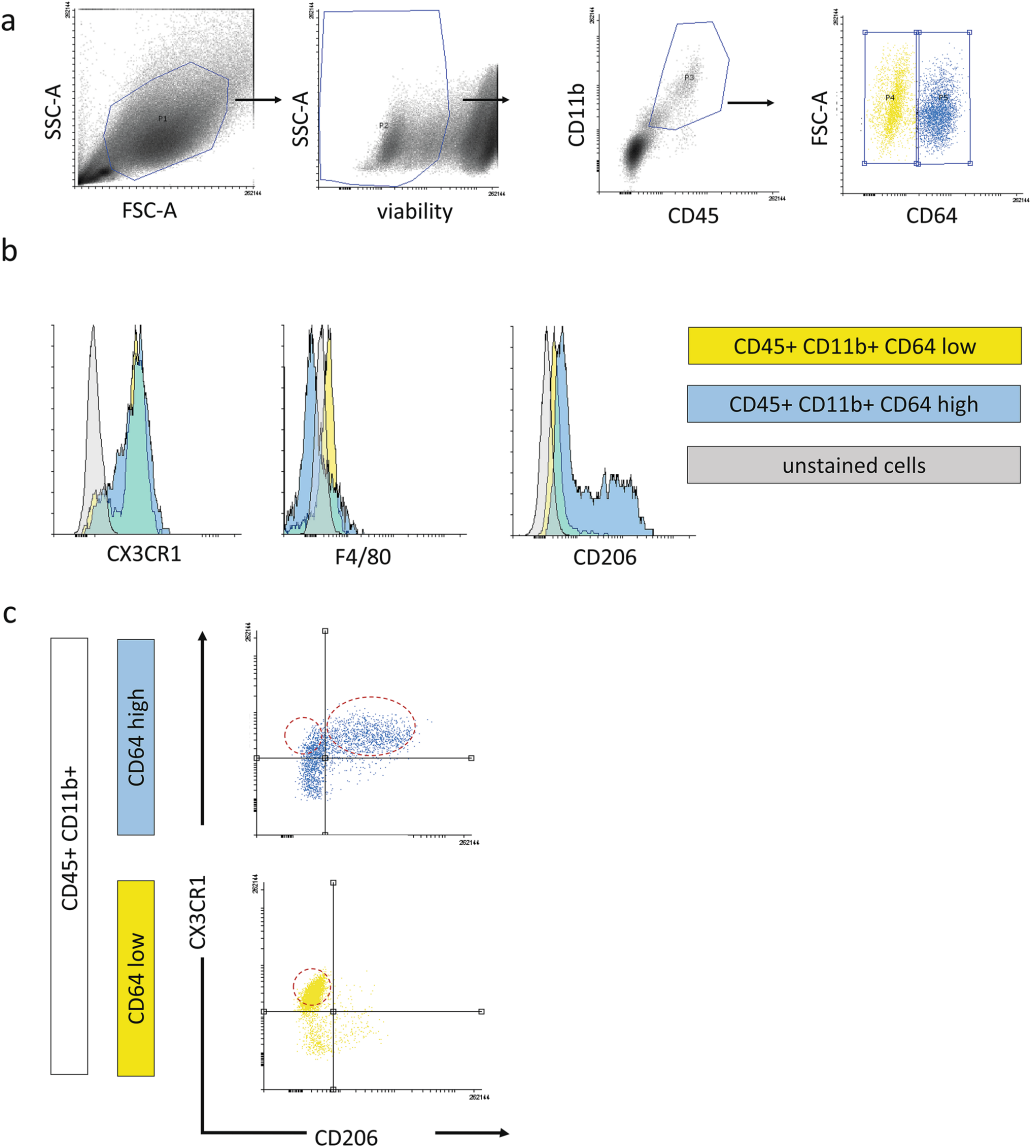
Flow cytometry analysis with identification of embryonic cTM subpopulations: CD64^low^; CD64^high^CD206-, and CD64^high^CD206+. **a** Gating strategy of E14 mouse cardiac tissue macrophages from single cell suspension. Firstly, dead cells were excluded. Within living cells [gate P2], the CD45-CD11b-double-positive population was selected [gate P3]. These cells were also identified as CD64+ with the significant differentiation on CD64^low^ [gate P4] and CD64^high^ [gate P5] subpopulations. **b** Representative histograms for CX3CR1, F4/80 and CD206 detected in CD45+CD11b+CD64^low^ population (yellow) and CD45+CD11b+CD64^high^ population (blue). Control staining is shown in gray. **c** Representative dot-plot analysis of pregated CD45+CD11b+CD64^low^ population (yellow) and CD45+CD11b+CD64^high^ population (blue) for CD206 and CX3CR3 expression. There is a clear differentiation on CD206+ and CD206- within CD64^high^ subpopulation in comparison with CD64^low^ subpopulation, which is CD206-. The red dashed lines indicate three selected cTM subpopulations: CD45+CD11b+CX3CR1+CD64^low^, CD45+CD11b+CX3CR1+CD64^high^CD206− and CD45+CD11b+CX3CR1+CD64^high^ CD206+.

We observed no significant differences between the two selected developmental stages regarding expression levels of selected markers. The gating strategy was first to select CD45/CD11b-double-positive and subsequently CD64-positive cells (a pan-macrophage marker) in FACS analysis, which resulted in defining of two populations: CD45+CD11b+CD64^low^ and CD45+CD11b+CD64^high^ (Fig. 7a). To evaluate our classification with regard to the origin of cTMs, we used anti-CX3CR1 antibodies. Therefore, within these populations, we were able to identify potentially yolk-sac-derived cTMs (CX3CR1^high^) and the fetal liver monocyte-derived cTMs (CX3CR1^low^) (Fig. 7b, c). Besides, there was a marked difference in CD206 expression within cTMs. Accordingly, a clear distinction between CD206+ cells and CD206- cells was observed within the CD64^high^ subpopulation, whereas the CD64^low^ subpopulation consisted mainly of CD206− (Fig. 7b, c). Moreover, the CD206+ cTM subpopulation also expressed theCX3CR1^high^ phenotype. These findings enabled us to distinguish the following macrophage subpopulations: CD45+CD11b+CX3CR1+CD64^low^, CD45+CD11b+CX3CR1+CD64^high^CD206− and CD45+CD11b+CX3CR1+CD64^high^ CD206+ (Fig. 7c).

The CD45+CD11b+CD64^low^ population was found to be F4/80-positive, whereas CD45+CD11b+CD64^high^ population was F4/80-negative (Fig. 7b). Thus, we did not detect any differences among cTMs based on their levels of CD11b and F4/80 expression. This finding is in contrast to earlier literature reports on macrophages in other embryonic tissues, where yolk-sac-derived macrophages had CD11b^low^ F4/80^high^ expression pattern, whereas fetal liver-derived macrophages were CD11b^high^ F4/80^low^ (Fig. 7b) (Epelman et al. 2014).

### Gene expression in cTM subpopulations

RT-PCR studies performed on hearts derived from E14 and E17-stage fetuses revealed differences in the level of mRNA expression for selected genes in the sorted subpopulations of cTMs (marked as P1, P2 and P3).

**Fig.8:**
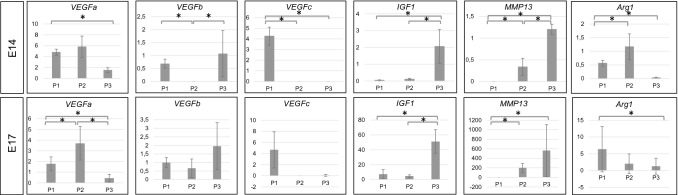
PCR analysis reveals differences among selected embryonic cTM subpopulation at two developmental stages (E14.0 and E17.0). The panel presenting expression levels of mRNAs for selected genes at two developmental stages: E14.0 and E17.0 within sorted cTMs subpopulations. P1: CD45+CD11b+F4/80+CD64^low^; P2: CD45+CD11b+F4/80+CD64^high^ CD206−; P3: CD45+CD11b+F4/80+CD64 ^high^ CD206+. Statistically significant differences (*p* value ≤ 0.05) are marked (*)

All subpopulations of cTMs at E14 and E17 stages expressed mRNA for *VEGFa*; however, the CD64^high^ CD206− (P2) exhibited the highest levels of this ‘classic’ proangiogenic factor-encoding mRNA (Fig. 8). mRNA for *VEGFb* was also detected in all subpopulations at two developmental stages, except the P2 exhibited the expression only at E17, being not detectable at E14. Interestingly, only the CD64^low^ (P1) subpopulation expressed mRNA for *VEGFc* at both developmental stages. Since *VEGFc* is a major lymphangiogenic factor, we suspect that the CD64^low^ macrophages might play a key role in lymphangiogenesis. mRNA levels for *IGF1* in the CD64^high^CD206+ (P3) subpopulation were considerably higher than in P1 and P2 subpopulations at both E14 and E17 (Fig. 8).

**Fig. 9:**
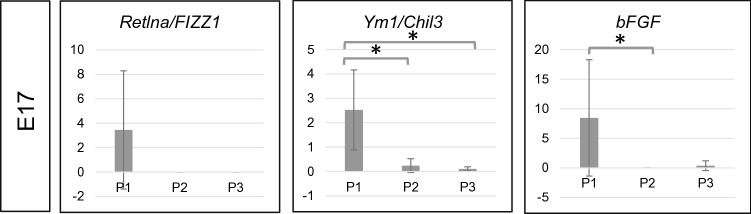
mRNAs for Retlna/FIZZ1, Ym1/Chil3 and bFGF are detectable only at the later developmental stage (E17.0). The panel presents expression levels of mRNAs for Retlna/FIZZ1, Ym1/Chil3 and bFGF at E17. These mRNAs were mainly expressed in the P1 subpopulation being undetectable at E14. P1: CD45+CD11b+F4/80+CD64+^low^; P2: CD45+CD11b+F4/80+CD64 + ^high^ CD206−; P3: CD45+CD11b+F4/80+CD64+^high^ CD206+. Statistically significant differences (*p* value ≤ 0.05) are marked (*)

Furthermore, the levels of mRNA for *Retlna/FIZZ1*, *Ym1/Chil3*, *bFGF*, and other factors with proangiogenic functions, were first detected at the later developmental stage (E17). The levels of these mRNAs molecules were highest in the CD64^low^ (P1) subpopulation. This suggests that macrophages mature and gain some markers during development (Fig. 9).

The levels of mRNA for factors involved in the extracellular matrix remodeling, such as *matrix metalloproteinase 13* (*MMP13*), *arginase 1* (*Arg1*) and *chitinase-like 3* (*Ym1/Chil3*), varied among subpopulations and developmental stages. At E14 and E17, the expression of mRNA for *MMP13* was present only in the CD64^high^ (P1 and P2) subpopulations and not detectable in the CD64^low^ (P1). mRNA levels for *MMP13* were higher in the CD64^high^CD206+ (P3) compared with the CD64^high^CD206− (P2) subpopulation (Fig. 8).

## Discussion

Previous studies on cTMs during heart development and postnatal stages have mainly focused on the origin of these cells and their gradual replacement by monocyte-derived macrophages after birth (Epelman et al. 2014; Lavine et al. 2014; Molawi et al. 2014). Here, we show a novel approach to characterize embryonic cTMs. We demonstrated that cTM constitutes a phenotypically diverse population in terms to their markers, location within cardiac tissue and the expression of certain genes. Since gene expressions of various mediators involved in angiogenesis, lymphangiogenesis and extracellular matrix remodeling were elevated in macrophage subsets, we presume these macrophage populations play roles in these processes during heart development.

Our findings indicate that the location of first cTMs in embryonic heart tissue is specifically restricted to the subepicardial space. This is consistent with other reports (Flaht et al. 2012; Stevens et al. 2016) and supports the theory that cardiac seeding by yolk-sac-derived macrophages depends on the epicardium formation and epicardium-dependent signaling including transcription factor WT1 (Stevens et al. 2016). Our results are also in line with the observation published by Balmer et al. on a spatiotemporal correlation between the development of the epicardium and the cardiac settlement of the first hematopoietic cells, which turned out to be cTMs (Balmer et al. 2014). The literature is inconsistent with regard to appearance of the first macrophages in the developing heart. According to Epelman et al. (2014), the first macrophages seed cardiac tissues at E9.5 (Epelman et al. 2014), whereas other authors detected first macrophages at E12.5 (Hoeffel et al. 2015). Here, we have shown first F4/80+ macrophages at E10 in whole-mount immunostained murine hearts. These cells are located in the subepicardial area, where the onset of angiogenesis and lymphangiogenesis also takes place. Proangiogenic and prolymphangiogenic functions of TMs have been described previously in pathological settings (Corliss et al. 2016; Ferraro et al. 2019; Ji 2012; Nucera et al. 2011). Our work shows that embryonic cTMs bearing mostly the Lyve-1 marker adhere to newly formed vessel walls forming direct cell-to-cell contacts. However, the phenotypes (i.e., expressed markers) of cTMs adhering to blood vessels do not differ from the phenotypes of cTMs adhering to lymphatics. A similar spatiotemporal relationship between tissue macrophages and blood vessels was previously described in detail in the developing hindbrain (Fantin et al. 2010) and in fetal mouse testis (DeFalco et al. 2014). A proangiogenic role of Lyve-1-positive macrophages by various activating pathways was also detected in the epididymal adipose tissue in rodents (Cho et al. 2007). As indicated in our study, the Lyve-1 antigen is present in the majority of embryonic cTMs and also in endothelial cells of early embryonic veins and lymphatic vessels. Our data are in line with previous reports, which presented Lyve-1 as a cTM-specific marker in adult hearts, in contrast to macrophages located in other tissues, such as the peritoneal, adipose and the pulmonary tissue, where Lyve-1 is expressed in lower number of macrophages (Pinto et al. 2012).

Apart from the subepicardially located cTMs, we found another population (CD68+) to be present in endocardial cushions and overlapping with regions of intensive apoptosis. This would indicate that some of embryonic cTMs are also phagocytically active in the area of the outflow tract shortening and valvular remodeling (Cheng et al. 2002; Vicente Steijn et al. 2018).

Our flow cytometry results highlight the heterogeneity of the cTM population. Embryonic cTMs proved to be CD11b+F4/80− or CD11b+F4/80+, which does not allow us to clearly differentiate between yolk-sac-derived and fetal liver-derived cTMs. Similarly, mixed origins of cells within selected subpopulation were defined based on the expression of CX3CR1. Therefore, using these markers, we could not observe any correlation between macrophages origin and their potential function. Our study defined novel subpopulations in fetal heart marked CD64^low^ and CD64^high^ within the population bearing CD45 and CD11b markers. We suggest that gaining the CD64 marker illustrates ‘maturation’ of macrophages during development, therefore reflecting the ‘mature’ (CD64 ^high^) and ‘immature’ (CD64^low^) subpopulations among cTMs. In fact, the CD64 receptor is used to identify macrophages, without a distinct difference in the level of expression of this marker (Leid et al. 2016). Additionally, the expression of a mannose receptor, CD206, typical for alternatively activated macrophages, could help to distinguish three main, novel embryonic cTMs subpopulations.

To further characterize cTMs, we selected a panel of mRNA for genes coding proteins involved in regulation of angiogenesis, lymphangiogenesis and extracellular matrix remodeling based on previous studies on adult cTMs (Pinto et al. 2012). Our results reveal significant differences in mRNA levels among sorted cTMs subpopulations, as well as between two developmental stages (E14 and E17). The CD64^high^ CD206 macrophages could be considered as a presumable proangiogenic subpopulation, since they express the highest levels of mRNA for *VEGFa*, a major angiogenic factor. Of note, this growth factor may also stimulate lymphangiogenesis by recruitment of monocytes/macrophages similar to some processes during inflammation (Cursiefen et al. 2004). On the other hand, the CD64^low^ subpopulation, expressing mRNA for the lymphangiogenic factor *VEGFc*, seems to be prolymphangiogenic. The crucial role of *VEGFc* in the formation and further sprouting of lymphatic capillaries in the heart is well established (Dashkevich et al. 2016; Henri et al. 2016). Moreover, *VEGFc* stimulates also angiogenesis (Chung et al. 2009; Tammela et al. 2011). Proangiogenic role of *VEGFc* in the fetal heart has been documented by Chen et al., who suggested a strong *VEGFc* functional orientation to only certain type of coronary vessels, i.e., sinus venosus-derived (Chen et al. 2014b), or its contribution to coronary artery stem formation, which indicates a localized activity of this growth factor (Chen et al. 2014a).

Interestingly, embryonic cTMs change their properties during heart development. The CD64^low^ subpopulation starts expressing the mRNA for *Retlna/FIZZ1*, *Ym1/Chil3*, *bFGF* in later developmental stages (starting atE17). *Ym1/Chil3* and *Retlna/FIZZ1* belong to the well-characterized markers of alternatively activated macrophages (M2), which take part in chronic inflammation (Raes et al. 2002). *Retlna/FIZZ1* (*a resistin-like molecule/found in inflammatory zones*) was firstly described in an experimental model of allergic pulmonary inflammation and then detected and studied also in the lung, liver and gut infested with helminths (Holcomb et al. 2000). *Retlna/FIZZ1* turned out to be a useful marker for Th2-dependent immune response (IL-4, IL-13), which is atypical for heart tissue (Liu et al. 2004). Moreover, *Retlna/FIZZ1* is strongly associated with fibrosis via an activation of alpha-smooth muscle actin and collagen type I expression in fibroblasts by *Notch1* signaling (Liu et al. 2004, 2009; Wynn and Barron 2010), its antiapoptotic effect on fibroblast (Chung et al. 2007) and interaction with extracellular matrix components (Malaguarnera 2006; Wynn and Barron 2010). The third gene whose expression was detected in later heart developmental stages was *bFGF*. As a ‘pleiotropic’ growth factor, *bFGF* is responsible for fibroblast and endothelial cells proliferation and migration (Okada-Ban et al. 2000). It also induces *VEGF* expression and affects endothelial cells by stimulating proteinase production and expression of cadherins and some angiogenic integrins (αvβ3) (Presta et al. 2005; Seghezzi et al. 1998).

Alternatively, CD64^high^ CD206+ subpopulation of cTMs could display antifibrotic activity, producing *MMP13*. As a member of matrix metalloproteinase family, this metalloprotease participates in collagen degradation and restructuring, although some studies indicate also its proinflammatory and profibrotic roles (Hattori et al. 2009; Uchinami et al. 2006; Wynn and Barron 2010). Although *MMP13* is commonly expressed during embryonic development mainly in the skeleton, initially no expression of *MMP13* was detected in other fetal tissues such as the skin, lungs, neural tissue, muscles and liver (Johansson et al. 1998). We cannot preclude the CD64^high^ CD206+ macrophages could participate in ECM remodeling via *MMP13* activation, but also in endothelial cell proliferation and migration mediated by *IGF1* (Bach 2015). Both *IGF1* and *IGF2* in embryonic CCR2− positive macrophages were identified as major potential mediators in coronary vessel remodeling (Leid et al. 2016; Wang et al. 2020).

Our studies do not indicate clearly that the M2-macrophage phenotype is typical for embryonic cTMs, as has been described in adult hearts at the steady state (Pinto et al. 2012). M2-macrophage markers revealed cTMs to be mainly Lyve1+ by our in situ studies, with only some of them CD206-positive, as confirmed by IF staining and flow cytometry analysis, whereas the CD163, a scavenger receptor of postnatal cTMs (Fabriek et al. 2005), was not expressed in prenatal cTMs. In addition, the expression of mRNA for the M2-macrophage-like genes *Retlna/FIZZ1* and *Ym1/Chil3* occurs later in development. Therefore, we showed that the resident cTMs mature and differentiate in normal-growing prenatal heart. Considering the presence of the CD206 antigen, we noticed some similarities between development, aging and pathological processes. Most macrophages present in the adult heart are of the M2 phenotype, with increasing number of CD206+ cells in the heart during physiological development and aging. Similarly, the number of the CD206+ (M2-like) macrophages increases after myocardial infarction (MI), especially locally in the infarct area at 7-day post-MI (Shiraishi et al. 2016). As a cell-surface scavenger receptor, CD206 mediates antigen processing, endocytosis and phagocytosis and plays a key role in the innate immune response (Gazi and Martinez-Pomares 2009). Apart from its antigen-binding function, CD206 together with other receptors acts as mediator in intracellular signaling pathways through cytokine system (Gazi and Martinez-Pomares 2009; Tachado et al. 2007).

Interestingly, a recent report provides a novel data on macrophage function in the cardiac conduction system, where macrophages, connected with cardiomyocytes via gap junctions, are an integral part of atrioventricular and sinoatrial node and other conduction system structures (Hulsmans et al. 2017; Leuschner and Nahrendorf 2020; Wang et al. 2020). This particular macrophage function during development or in conduction disorders has not been yet, however, elucidated.

Considering cTM location, immunophenotype and gene expression analysis, we suggest that embryonic cTMs could play various roles during heart development. Based on altered mRNA levels of several mediators involved in angiogenesis, lymphangiogenesis and extracellular matrix remodeling in three selected populations, we can presume that these macrophage subsets may play relevant roles in these processes. Our findings require further studies, to define actual macrophage activities by analysis of their proteome profile. Moreover, since some similarities are emerging between potential embryonic macrophages functions and a significant role of macrophages in pathological stages, the data presented in our studies reveal a new area for further research on cardiac tissue macrophages.
